# RAGE Cytosolic Partner Diaph1 Does Not Play an Essential Role in Diabetic Peripheral Neuropathy Progression

**DOI:** 10.3390/cells14201635

**Published:** 2025-10-21

**Authors:** Kamila Zglejc-Waszak, Bernard Kordas, Agnieszka Korytko, Andrzej Pomianowski, Bogdan Lewczuk, Joanna Wojtkiewicz, Krzysztof Wąsowicz, Izabella Babińska, Konark Mukherjee, Judyta Juranek

**Affiliations:** 1 Department of Anatomy and Histology, Faculty of Medicine, Collegium Medicum, University of Warmia and Mazury in Olsztyn, 10-085 Olsztyn, Poland; 2 Department of Human Physiology and Pathophysiology, Faculty of Medicine, Collegium Medicum, University of Warmia and Mazury in Olsztyn, 10-085 Olsztyn, Polandjoanna.wojtkiewicz@uwm.edu.pl (J.W.); 3 Internal Medicine Department, Faculty of Veterinary Medicine, University of Warmia and Mazury in Olsztyn, 10-719 Olsztyn, Poland; 4 Department of Histology and Embryology, Faculty of Veterinary Medicine, University of Warmia and Mazury in Olsztyn, 10-719 Olsztyn, Poland; lewczukb@uwm.edu.pl; 5 Department of Pathophysiology, Forensic Veterinary Medicine and Administration, Faculty of Veterinary Medicine, University of Warmia and Mazury in Olsztyn, 10-719 Olsztyn, Poland; 6 Department of Genetics and VSRC, University of Alabama at Birmingham, Birmingham, AL 35233, USA

**Keywords:** cytoskeleton, type 1 diabetes, neuropathy, sciatic nerve, Diaph1, RAGE

## Abstract

**Highlights:**

**What are the main findings?**

- Global deletion of Diaph1 disrupts beta-actin polymerization in the sciatic nerve during type 1 diabetes.
- We observed decreased nerve conduction velocity and abnormalities in sciatic nerve morphometry in diabetic Diaph1 knockout diabetic mice.

**What is the implication of the main finding?**

- Deletion of Diaph1 is insufficient to halt the progression of diabetic peripheral neuropathy in mice.
- However, we cannot unequivocally rule out that Diaph1 is an important switch role in the RAGE pathway and diabetic peripheral neuropathy.

**Abstract:**

Receptor for advanced glycation end-products (RAGE) activation by hyperglycemia-induced AGE (advanced glycation end-products) accumulation is likely to play a crucial role in the development of complications such as diabetic peripheral neuropathy (DPN). RAGE signaling is mediated via its cytosolic tail. Through its cytosolic tail, RAGE recruits diaphanous-related formin 1 (Diaph1), a protein involved in actin filament organization. Disruption of RAGE–Diaph1 interactions using small molecules alleviates diabetic complications in mice; however, the role of Diaph1 in DPN progression has not been rigorously tested. In this study, we employed a Diaph1 knockout mouse (DKO) to investigate the role of Diaph1 in DPN progression. Herein, we demonstrate that, at the systemic level, CRISPR deletion of Diaph1 fails to ameliorate diabetes-induced weight loss in mice. Within the sciatic nerve (SCN), the lack of Diaph1 failed to prevent hyperglycemia-induced loss of β-actin in the nerve fibers. At a morphological level, the lack of Diaph1 leads to a partial rescue in DPN. While we observed improvements in axonal and fiber diameters in diabetic DKO mice, the g-ratio (an indicator of myelination) and myelin invaginations displayed incomplete rescue. Furthermore, the lack of Diaph1 failed to rescue motor or sensory nerve conduction defects resulting from hyperglycemia over 6 months. Overall, our data thus indicate that the complete loss of Diaph1 is insufficient to halt the progression of DPN. However, across a range of parameters including blood glucose levels, body weight measurements, axon and fiber diameters, and nerve conduction velocity, DKO diabetic mice show improvement when compared to wild-type diabetic mice.

## 1. Introduction

Type 1 diabetes (T1D) is a metabolic disorder of multifactorial origins [1]. Treating T1D can be a challenging and lengthy process [1,2,3,4,5,6,7]. T1D can lead to various complications, including damage to blood vessels, microvessels, and peripheral nerve fibers, ultimately resulting in diabetic peripheral neuropathy (DPN) [1,3]. Several pathways have been suggested to be crucial for diabetic neuropathy, including polyol, hexosamine, protein kinase C (PKC), and advanced glycation end-products (AGEs) pathways. Growing evidence suggests that the pathological changes observed in diabetic neuropathy might result from several concomitant factors such as increased inflammation, increased oxidative stress, protein glycation, and axonal transport alteration [3,4,6]. The association of chronic hyperglycemia with diabetes mellitus intuitively suggests that signaling mediated by AGEs is involved [1,2,5]. Furthermore, the constellation of cellular mechanisms mentioned above points to a ubiquitously distributed signaling mechanism, and suggests RAGE (receptor of AGEs) and its cytosolic partner Diaph1 (mammalian diaphanous, mDia1) as potential molecular contributors and therapeutic targets in DPN prevention and treatment [1,2,3,4,5,6]. Our previous studies have shown that the RAGE-Diaph1 pathway is active in the sciatic nerve during T1D [2,5,8,9,10]. We observed that the amount of RAGE protein was increased while levels of both Diaph1 mRNA and protein were decreased in the sciatic nerves of T1D patients [2,5,8,9,10]. Deletion of Diaph1 has been shown to be potentially beneficial in mouse models of atherosclerosis, cardiac neuropathy, and nephropathy [7,11,12,13,14,15]. However, it is still unclear whether Diaph1 contributes directly to the exacerbation of DPN. It is also unclear whether any global increase or decrease in Diaph1 expression might alleviate certain components of DPN.

Our present study using a Diaph1 knockout mouse line indicates that Diaph1 does not play a major role in DPN progression. We demonstrate that the deletion of Diaph1 does not reverse any molecular, morphological, or structural changes in a DPN mouse model.

## 2. Materials and Methods

### 2.1. Animals

A Diaph1 knock-out (DKO) mouse line was generated using the CRISPR/Cas9 method (International Institute of Molecular and Cell Biology in Warsaw, Poland). To verify Diaph1 deletion, polymerase chain reaction (PCR)-based genotyping was performed using specific Diaph1 primers (Forward: GCATTGCTGTCTCTTACACA, Reverse: TCAACTTAGGAGACCACACA). Three Diaph1 products, at 504 bp and 313 bp and 201 bp, confirmed DKO status (Figure S1). Mice were housed in ventilated rooms under a light–dark period (12 h-12 h) at 21 °C with free access to food (Labofeed B standard) [2,8]. Due to the protective effect of estrogen, only male mice were used in the study. Eight-week-old male mice (C57BL/6 (Wild type—WT), DKO (C57BL/6 background) were randomly divided into experimental groups per defined time points. T1D was induced by intraperitoneal injection of streptozotocin (STZ, 50 mg/kg; Sigma-Aldrich, Saint Louis, MO, USA) for five consecutive days [2,8]. Mice were sacrificed 24 weeks post the last STZ injection (six months of T1D). Samples for morphometric and immunofluorescence staining were stored in cryoprotective buffer [2]. The study was approved by the Local Ethics Committee of Experiments on Animals in Olsztyn, Poland; decision no. 57/2019. The risk of animal suffering was minimized.

### 2.2. Immunofluorescence Staining

The staining protocol was described in detail in previous publications [2,8,9,10,16]. We used CFL (cofilin; CFL1 + 2; Table 1) antibody specific to a protein found in muscle (CFL2) and nerve tissue (CFL1). The levels of protein expression were quantified using a fluorescence microscope (Olympus IX83, Tokyo, Japan) and ImageJ v. 1.54k [2,8,9,10,16]. Negative control confirmed the specificity of antibodies (Figure S2).

### 2.3. Morphometric Studies

The analysis involved two distinct genotypes, WT and DKO, with each genotype divided into two experimental groups, control and diabetes, to assess the impact of diabetic conditions on nerve pathology [2,8,9,10,16].

### 2.4. Nerve Conduction Velocity (NCV)

The study was described in detail by Jaroslawska and co-workers [8] and by Schulz et al. [17]. All animals were intraperitoneally anesthetized with a mixture of ketamine (100 mg/kg) and xylazine (10 mg/kg) diluted in saline [2,8]. We confirmed sufficient anesthesia by testing sensitivity for low-grade pain.

### 2.5. Statistical Analysis

Statistical results were visualized using GraphPad Prism 9.1.0. (San Diego, CA, USA). Statistical analyses were performed in Statistica v. 13 (StatSoft Inc.). Before choosing a statistical test, we performed a Shapiro–Wilk normality test. Based on the test results, we selected either one-way ANOVA or the Kruskal–Wallis test. A value of *p* ≤ 0.05 was considered statistically significant [2,18].

## 3. Results

### 3.1. Impact of Diaph1 Deletion on Weight and on Blood Glucose Levels

**Figure 1:**
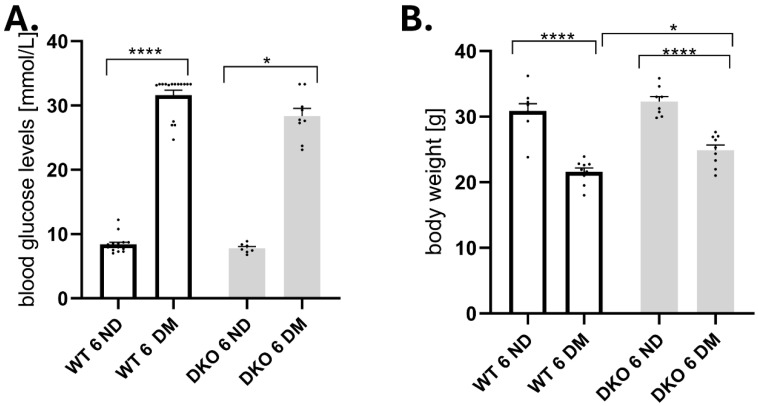
Streptozotocin (STZ)-induced diabetes-related phenotypic characteristics in wild-type (WT) and Diaph1 knockout (DKO) mice. Blood glucose (**A**) and body weight (**B**) differ at three and six months after STZ administration. Data are expressed as means ± SEM. * *p* ≤ 0.05; **** *p* ≤ 0.0001. Numbers of individual animals in the study are indicated in the figure as individual data points. Abbreviations: DM—diabetic mellitus (type 1 diabetes), ND—non-diabetic (control).

We measured glucose levels in four groups of mice (WT 6 ND, WT 6 DM, DKO 6 ND, DKO 6 DM). Blood glucose levels were already more than twice as high after six months in the T1D groups (WT and DKO) compared to their control groups (respectively, WT 6 ND and DKO 6 ND; Figure 1A). DKO mice developed hyperglycemic levels quickly and to the same level as WT mice (*p* ≤ 0.0001 and *p* ≤ 0.05, respectively; Figure 1A). Rapid weight loss is often a sign of T1D. As diabetes progressed, we observed weight loss in both WT and DKO experimental groups (*p* ≤ 0.0001, Figure 1B); however, weight loss was more pronounced in WT mice (*p* ≤ 0.05, Figure 1B).

### 3.2. Amounts of ACTB, PFN1, CFL, and RhoA upon Deletion of Diaph1

**Figure 2:**
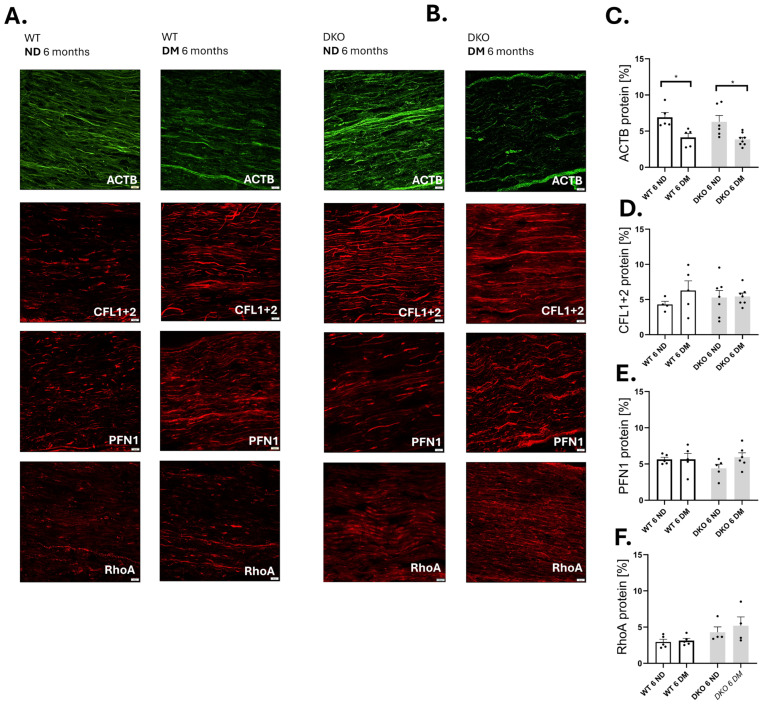
Changes in actin and its regulator molecules in diabetic DKO mice. (**A**) Immunostaining of ACTB, PFN1, CFL, and RhoA proteins in WT mouse sciatic nerve. (**B**) Immunoreactivity of ACTB, PFN1, CFL, and RhoA upon deletion of Diaph1 in T1D mice. Changes in amounts of ACTB (**C**), PFN1 (**D**), CFL1 + 2 (**E**), and RhoA (**F**) in mice three and six months after STZ administration. Scale bar = 20 μm. Supplementary Figure S2 shows negative control. An additional comparison of shape features can be found in Supplementary Figure S3. In Supplementary Figure S3, yellow color indicates colocalization of ACTB-PFN1 and ACTB with CFL1 + 2. Data are expressed as means ± SEM. * *p* ≤ 0.05. Numbers of individual animals in the study are indicated in the figure as individual data points. Abbreviations: DM—diabetic mellitus (type 1 diabetes), ND—non-diabetic (control).

Diaph1 is known for its role in actin remodeling within cells. This prompted us to investigate the levels of proteins involved in actin cytoskeleton dynamics, such as β-actin, profilin 1, cofilin, and Ras Homolog Family Member A (ACTB, PFN1, CFL, and RhoA), upon Diaph1 deletion in the mouse sciatic nerve [4,19]. Our findings revealed that all studied proteins were present in the sciatic nerve in both DKO and WT mice (Figure 2A,B). The amount of ACTB was decreased in sciatic nerve harvested from 6-month diabetic mice (both WT and DKO) compared to respective control groups (*p* ≤ 0.05, Figure 2C). We did not observe differences in amounts of CFL and PFN1 in the sciatic nerve harvested from WT mice during the course of the disease (*p* ≥ 0.05, Figure 2D,E). RhoA protein patterns in DKO and WT mice were also indistinguishable (*p* ≥ 0.05, Figure 2F). We assumed, then, that global deletion of Diaph1 does not affect actin cytoskeleton dynamics in the sciatic nerve during diabetes.

### 3.3. Morphological Alterations in Sciatic Nerve

**Figure 3:**
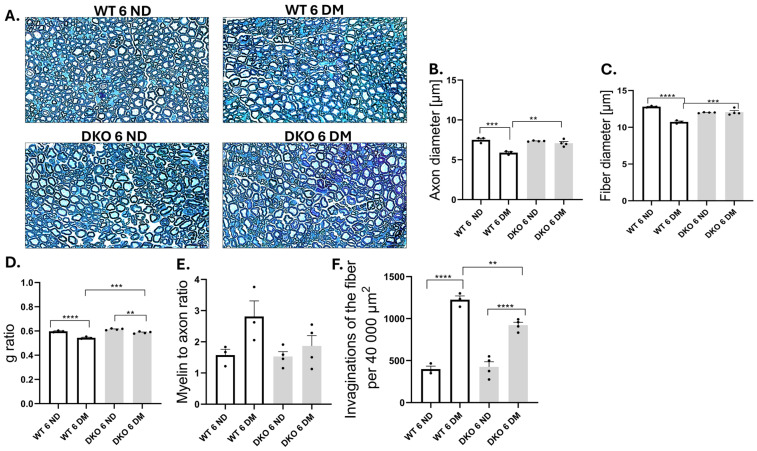
Morphological changes in SCN in diabetic DKO mice. (**A**) Representative images of sciatic nerve sections from control and experimentally treated mice. Fiber diameter–axon diameter with myelin thickness. (**B**–**F**) Numbers of individual animals in the study are indicated in the figure as individual data points. Data are expressed as means ± SEM. ** *p* ≤ 0.01; *** *p* ≤ 0.001; **** *p* ≤ 0.0001. Abbreviations: DM—diabetic mellitus (type 1 diabetes), ND—non-diabetic (control).

DPN progression is often associated with morphological changes in the nerves. Morphometric abnormalities were quantified within the specified region of interest (ROI) (sciatic nerve cross-section area of 40,000 μm^2^; Figure 3A). We observed a similar pattern of changes in both axon and fiber diameter (Figure 3B,C). Axon as well as fiber diameters were smaller in diabetic WT mice compared to controls (Figure 3B,C; *p* ≤ 0.001, *p* ≤ 0.0001, respectively). A similar reduction in axon and fiber diameter (total diameter of the axon plus its myelin sheath) was not observed in the DKO mice, indicating that Diaph1 may play a role in this process (Figure 3B,C, *p* ≥ 0.05). G-ratio is the ratio of axonal diameter to the fiber (axon + myelin) diameter and measure of myelin thickness. We observed a difference in g-ratio between control and diabetic groups in both WT and DKO mice (Figure 3D, *p* ≤ 0.001, *p* ≤ 0.01, respectively). T1D increases the number of invaginated fibers (myelin sheath forms inward) in mice (Figure 3F, *p* ≤ 0.0001). However, there were more pronounced losses in axon and fiber diameters and g-ratios in WT T1D mice, compared to DKO T1D mice (*p* ≤ 0.01, *p* ≤ 0.001, *p* ≤ 0.001; Figure 3B,C). Compared to diabetic WT mice, diabetic DKO mice had fewer fiber invaginations; however, the lack of Diaph1 did not abolish this process, and neither did it affect the myelin-to-axon ratio (Figure 3E, *p* ≥ 0.05). However, the growth in invaginations of the fiber was more pronounced in WT T1D mice than in DKO T1D mice (*p* ≤ 0.01, Figure 3F). Further study is necessary to confirm and explain our results.

### 3.4. NCV Under Diaph1 Control

**Figure 4:**
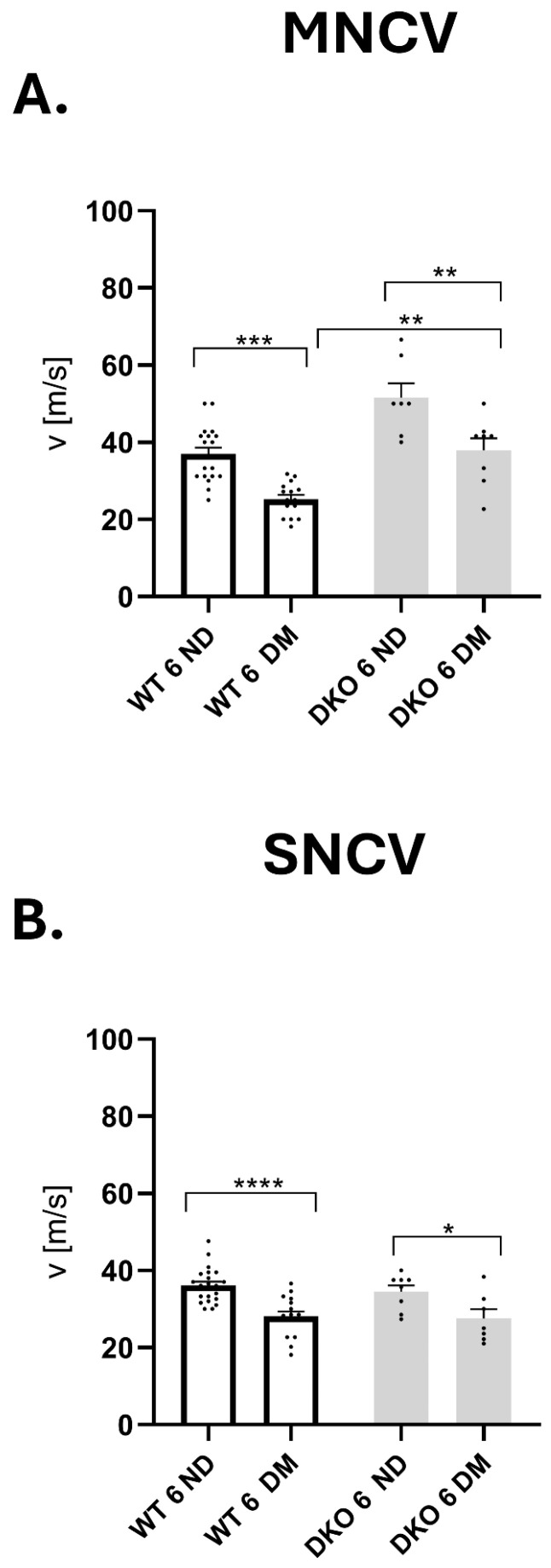
Functional changes in SCN in diabetic DKO mice. Motor (**A**) and sensory (**B**) nerve conduction velocity (MNCV and SNCV, respectively) measured three and six months after STZ administration. Data are expressed as means ± SEM. Numbers of individual animals in the study are indicated in the figure as individual data points. Data are expressed as means ± SEM. * *p* ≤ 0.05; ** *p* ≤ 0.01; *** *p* ≤ 0.001; **** *p* ≤ 0.0001. Abbreviations: DM—diabetic mellitus (type 1 diabetes), ND—non-diabetic (control).

NCV is the gold-standard method to confirm DPN, and significantly decreased NCV is a hallmark of DPN. The changes caused by T1D in NCV are similar in both sensory (SNCV) and motor (MNCV) fibers in WT mice (Figure 4A,B).

Nevertheless, similarly to WT mice, in diabetes, DKO mice displayed a similar reduction in MNCV compared to controls (*p* ≤ 0.001, *p* ≤ 0.01, respectively; Figure 4A). However, the reduction in MNCV was more pronounced in WT T1D mice than in DKO T1D mice (*p* ≤ 0.01, Figure 4A).

The results of SNCV examination were similar for WT and DKO in control and diabetic groups (*p* ≤ 0.0001, *p* ≤ 0.05, respectively, Figure 4B). Our results confirmed the development of DPN in both WT and DKO mice.

## 4. Discussion

Activation of RAGE signaling in chronic hyperglycemia promotes the development of diabetic complications, including DPN. Intracellular signaling via RAGE is thought to be dependent on Diaph1, which is recruited to the cytosolic tail of RAGE. Manigrasso and co-workers [13,14] reported that small-molecule antagonism of the RAGE-Diaph1 interaction may attenuate but not abolish diabetic complications in mice. This observation suggests that RAGE-Diaph1 signaling contributes to DM-associated morbidities such as DPN. Despite this indirect evidence, the role that Diaph1 plays in the progression of DPN has not been adequately examined.

To understand the overall role that Diaph1 may play in DPN progression, we employed Diaph1-null mice in our study. Moreover, because Diaph1 may also affect insulin resistance, and given that our mice had a global deletion of Diaph1, we aimed to determine whether their overall health was impacted in our T1D model. Our previous studies indicated that levels of low-density lipoprotein (LDL), high-density lipoprotein (HDL), total cholesterol, and triglycerides decreased in the T1D mouse model [8]. Therefore, instead of focusing on lipid profiles, we assessed the body weights of the mice. Our data indicates that deletion of Diaph1 is not sufficient to abrogate the weight loss that accompanies T1D.

Diaph1 was first discovered in 1997 and was then identified as a key regulator of actin dynamics [19,20]. The Formin Homology 1 (FH1) domain of Diaph1 can bind the actin–profilin complex, thereby promoting actin elongation [2,4,21]. In turn, the FH2 domain of Diaph1 can remodel and dimerize actin [22,23]. We previously showed that Diaph1 was important in nerve fibers and co-localized with β-actin as well as PFN1 in the sciatic nerve [2]. CFL acts as an antagonist of PFN1 because it is responsible for actin depolymerization. Thus, CFL, together with PFN1, regulates the dynamics of the actin cytoskeleton [4,22,23,24,25]. Diaph1 typically remains in an autoinhibited form, but it may be activated by RhoA, with which it co-localizes [23]. RhoA abolishes the autoinhibitory effects of the FH3 and DAD domains within the Diaph1 molecule. The dynamics and activities of Diaph1 are still unclear. However, it is indisputable that Diaph1 is an intracytoplasmic RAGE ligand. Based on the literature, we inquired whether loss of Diaph1 might affect the level of β-actin or its regulator under hyperglycemic conditions. Surprisingly, loss of Diaph1 is not enough to reverse the reduction in β-actin levels in nerve fibers induced by diabetes. Thus, the reduction in actin levels may not be a direct consequence of aberrant Diaph1 signaling in DPN. Furthermore, although we observed partial rescue in the morphology of SCN in diabetes with loss of Diaph1, this rescue was limited to diameters of nerve fibers and axons. The changes in myelin remained. In line with these biochemical and morphological findings, the absence of Diaph1 was insufficient to reverse the progress of DPN. Notably, after 6 months of diabetes, DKO mice displayed impaired MNCV and SNCV, just like WT mice. In fact, DKO mice displayed the same reductions in MNCV and SNCV as WT mice. However, the depth of MNCV and SNCV impairment was stronger in WT T1D mice than in DKO T1D mice. Our results confirmed the notion that global deletion of Diaph1 may attenuate but not abolish diabetic complications in mice [13,14].

We noticed that malfunctions in the expression of molecules involved in the cytoskeleton structure of nerve fiber in peripheral nerves of DKO mice may play an important role in progression of DPN. Moreover, the first symptoms of DPN may be visible in altered actin cytoskeleton dynamics under a high glucose environment. Nevertheless, we should not overlook the roles played by Diaph1 and other cytoskeleton proteins in the progression of DPN. Our findings raise an important question: how, then, does Diaph1 contribute to DPN? Here, we briefly consider the possibilities. It is possible that during T1D RAGE may interact with other molecules like TIRAP which also play a role in DPN. RAGE activation often induces kinase activity such as MAPK that may or may not be directly dependent on Diaph1 [1,2,3]. Finally, in the constitutive Diaph1 knockout mice, there could be compensatory changes to support the RAGE signaling. It is also possible that DPN is affected by Diaph1-independent metabolic changes arising from reduced insulin signaling, changes in glucose homeostasis, aberrant endocrine functioning, and impaired immune responses [26,27,28,29,30,31,32]. However, other mechanisms regulating the dynamics of the actin cytoskeleton have not been investigated in recent studies. Overall, our study is the first to examine DPN progress in the absence of Diaph1. Our conclusion is that Diaph1 may not be essential for DPN progression. Diaph1 may be crucial in obesity and lipid disorders, but our results do not confirm its therapeutic potential in DPN [26,27,28,29,30,31,32].

Diaph1 is an intracellular RAGE ligand [13,14,27,28,29,30,31,32]. It appears to be a key switch in the RAGE pathway. However, RAGE has multiple extracellular ligands. These ligands may play key roles in the RAGE pathway and in DPN progression [1,2,3]. Soluble RAGE (sRAGE) refers to circulating forms of RAGE that lack the transmembrane domain and act as decoy receptors [1]. sRAGE can bind RAGE ligands, preventing their interaction with cell-bound RAGE and thus modulating actin polymerization and tissue damage during DPN. Our previous studies indicated high levels of sRAGE in the plasma of STZ mice [8]. Interestingly, the results indicated low tissue expression of extracellular RAGE ligands [8] and thus slow progression of DPN. This phenomenon may confirm that Diaph1 may not be a key switch in the RAGE pathway during DPN [33,34,35].

Our studies indicated that targeting the intracellular RAGE domain/tail may not be an effective strategy to block RAGE signaling in DPN. However, further investigation of the differences between pharmacological inhibition (partial, local, and reversible) and genetic deletion (global, permanent) may explain the discrepancy between this study and earlier positive reports. Nevertheless, our previous studies indicated that amounts of Diaph1 protein in the sciatic nerves of diabetic mice were lower than in control groups [2,9]. We speculated that the observed differences in MNCV vs. SNCV might suggest a more ambiguous role of Diaph1 during DPN. However, DKO diabetic mice show improvement in all studied parameters when compared to WT diabetic mice. Thus, further studies are necessary to clarify the role of Diaph1 during neurological complications.

## 5. Conclusions

Hyperglycemia-induced malfunctions in the actin cytoskeleton structure and the actin dynamics process remain accepted mechanisms for the pathogenesis of DPN in WT and DKO mice. Collectively, the results of the present study suggest that blockade of Diaph1 may not be a key adjunctive strategy in therapeutic approaches to DPN. Our findings may reveal novel, Diaph1-independent mechanisms regulating actin cytoskeleton dynamics during DPN. However, further studies are necessary to clarify the roles played by Diaph1 in the RAGE signaling pathway and the progression of DPN. This study may indicate that therapies during DPN should take into account a number of parameters including the RAGE signaling pathway, Diaph1, sRAGE, and proteins involved in actin polymerization and inflammation.

## Figures and Tables

**Table 1:** Antibodies used for Western blot analysis and immunofluorescence staining.

Primary Antibodies					
Antigen	Code	Species		Working Dilution	Supplier
Immunofluorescence staining					
Sciatic nerve					
ACTB	251006	Chicken		1:200	Synaptic System, Germany
PFN1	Ab232020	Rabbit		1:100	Abcam, CA, UK
CFL1 + 2	Ab131519			1:100	
RhoA	Ab187027			1:100	
Secondary antibody					
Immunofluorescence staining					
Reagents	Code		Working dilution		Supplier
Goat anti-Rabbit IgG (H + L) Highly Cross-Adsorbed Secondary Antibody, Alexa Fluor™ Plus 594	A32740		1:2000		ThermoFisher, Oxford, UK
Goat anti-Chicken IgY, Alexa Fluor 488	A-11039				

## Data Availability

All relevant data are within the manuscript and Supporting Information.

## Supplementary Materials

The following supporting information can be downloaded at: https://www.mdpi.com/article/10.3390/cells14201635/s1, Figure S1. Picture of genotyping with various alleles. Analysis of PCR product patterns was carried out by gel electrophoresis in 1.5% agarose. Figure S2. Negative control confirmed the specificity of antibodies. Figure S3. Co-localization of ACTB-PFN1 and ACTB-CFL1+2 in mice sciatic nerve of T1D. Images were taken under 40× objective with 0.6 numerical aperture (40×/0.6). Scale bar = 20 μm. Abbreviations: WT—wild-type, DKO—Diaphanous knockout, DM—diabetic mellitus (type 1 diabetes), ND—non-diabetic (control).

## Abbreviations

The following abbreviations are used in this manuscript:

RAGE	Receptor for advanced glycation end-products
AGE	Advanced glycation end-products
CFL	Cofilin
DPN	Diabetic peripheral neuropathy
Diaph1	Diaphanous-related formin 1
DKO	Diaph1 knockout mouse
FH1	Formin homology 1
HDL	High-density lipoprotein
LDL	Low-density lipoprotein
mDia1	Mammalian diaphanous
MNCV	Motor nerve conduction velocity
NCV	Nerve conduction velocity
PKC	Protein kinase C
ROI	Region of interest
SNCV	Sensory nerve conduction velocity
STZ	Streptozotocin
T1D	Type 1 diabetes
WT	Wild type
