# Supplementary material for: RAGE Cytosolic Partner Diaph1 Does Not Play an Essential Role in Diabetic Peripheral Neuropathy Progression
Source: Cells. 2025 Oct 21;14(20):1635. doi: 10.3390/cells14201635 (PMC12563116; doi:10.3390/cells14201635)
Supplement: Supplementary file 1 [file cells-14-01635-s001.zip › cells-3887772-supplementary.pdf]

Analysis of PCR products pattern was done by gel electrophoresis in 1,5% agarose/TAE

Representative genotyping picture before and after BamHI digestion:

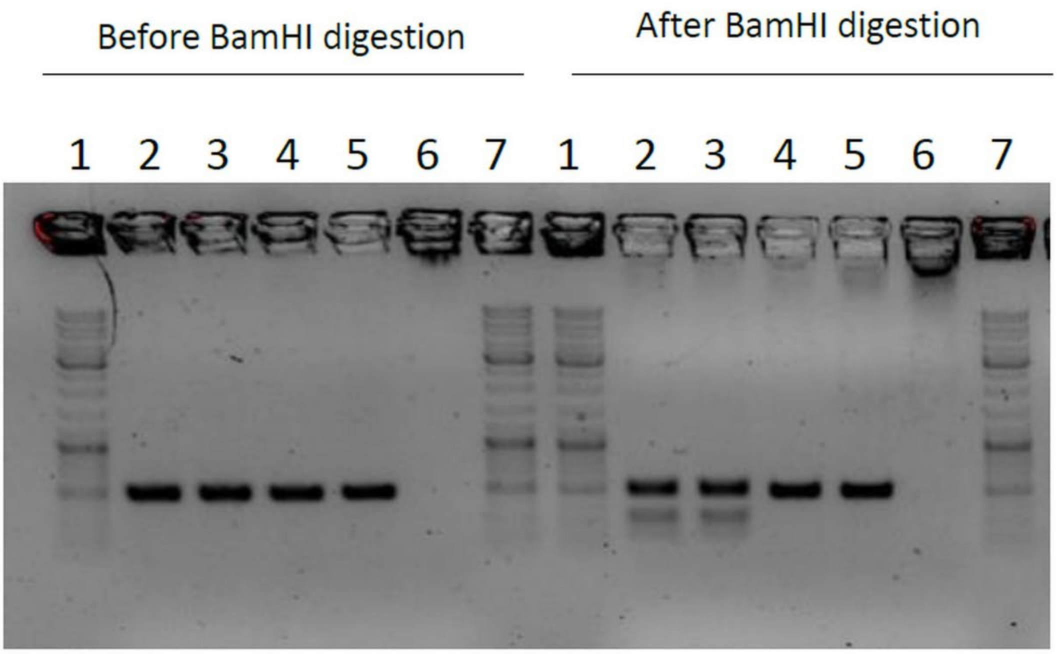

1. Gene Ruler Mix Ladder
2. Diaph1(insKO_casette/wt)
3. Diaph1(insKO_casette/wt)
4. WT
5. WT
6. No template control
7. Gene Ruler Mix Ladder

Figure S1. Picture of genotyping with various alleles. Analysis of PCR product patterns was carried out by gel electrophoresis in 1.5% agarose.

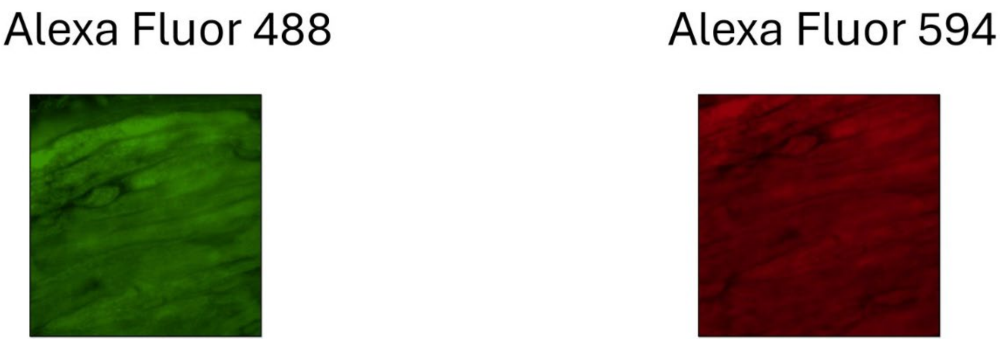

Figure S2. Negative control confirmed the specificity of antibodies.

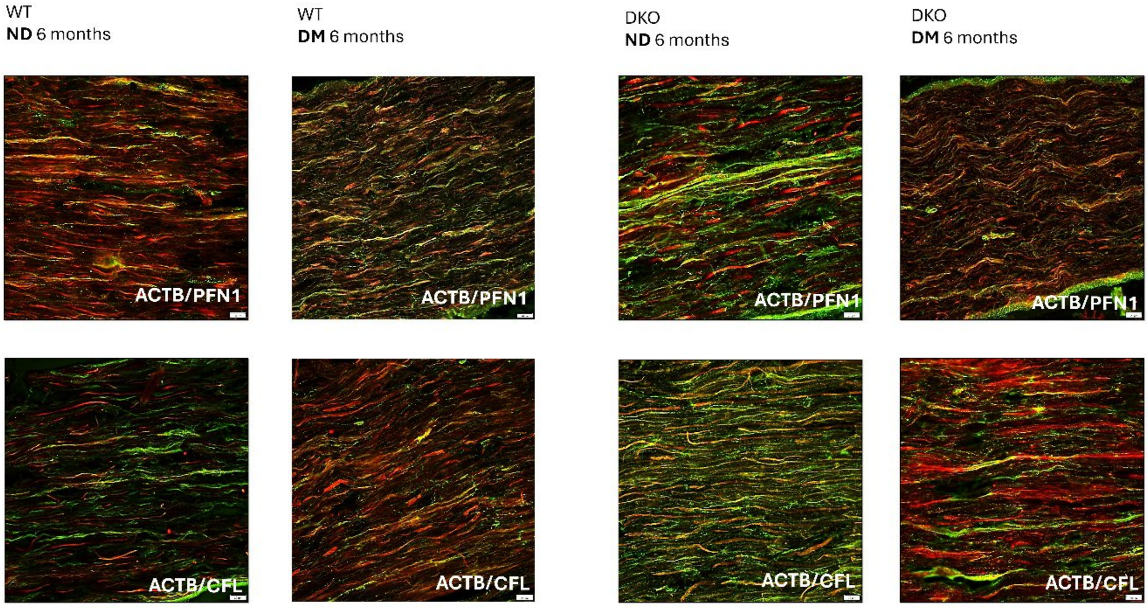

Figure S3. Co-localization of ACTB-PFN1 and ACTB-CFL1+2 in mice sciatic nerve of T1D. Images were taken under 40× objective with 0.6 numerical aperture (40× /0.6). Scale bar = 20 μm. Abbreviations: WT – wild-type, DKO – Diaphanous knockout, DM – diabetic mellitus (type 1 diabetes), ND – non-diabetic (control).
